# Mobile PCR-based surveillance for SARS-CoV-2 to reduce visiting restrictions in nursing homes during the COVID-19 pandemic: a pilot study

**DOI:** 10.1007/s15010-021-01716-4

**Published:** 2021-10-20

**Authors:** Jannik Stemler, Theresa Kramer, Vassiliki Dimitriou, Ulrike Wieland, Sofie Schumacher, Rosanne Sprute, Max Oberste, Gerhard Wiesmüller, Harald Rau, Sally Pieper, Ullrich Bethe, Clara Lehmann, Martin Hellmich, Florian Klein, Georg Langebartels, Oliver A. Cornely

**Affiliations:** 1 Faculty of Medicine and University Hospital Cologne, Department I of Internal Medicine, Excellence Center for Medical Mycology (ECMM), University of Cologne, Cologne, North Rhine-Westphalia Germany; 2 Faculty of Medicine and University Hospital Cologne, Chair Translational Research, Cologne Excellence Cluster On Cellular Stress Responses in Aging-Associated Diseases (CECAD), University of Cologne, Herderstrasse 52, 50931 Cologne, North Rhine-Westphalia Germany; 3 German Centre for Infection Research (DZIF), Partner Site Bonn-Cologne, Cologne, North Rhine-Westphalia Germany; 4 Faculty of Medicine and University Hospital Cologne, Institute of Virology, University of Cologne, Cologne, North Rhine-Westphalia Germany; 5 Faculty of Medicine and University Hospital Cologne, Institute of Medical Statistics and Computational Biology (IMSB), University of Cologne, Cologne, North Rhine-Westphalia Germany; 6 Department of Public Health, City Council of Cologne, Cologne, North Rhine-Westphalia Germany; 7 Department of Social Affairs, Health and Environment, City Council of Cologne, Cologne, North Rhine-Westphalia Germany; 8 Faculty of Medicine and University Hospital Cologne, Department I of Internal Medicine, University of Cologne, Cologne, North Rhine-Westphalia Germany; 9 Faculty of Medicine and University Hospital Cologne, Department for Clinical Affairs and Crisis Management, University of Cologne, Cologne, North Rhine-Westphalia Germany; 10 Faculty of Medicine and University Hospital Cologne, Clinical Trials Centre Cologne (ZKS Köln), University of Cologne, Cologne, North Rhine-Westphalia Germany

**Keywords:** COVID-19 pandemic, Nursing home, Surveillance, Testing on site, SARS-CoV-2 transmission

## Abstract

**Purpose:**

Residents in nursing homes for the elderly (NH) are at high risk for death from COVID-19. We investigated whether repeated non-mandatory RT-PCR SARS-CoV-2 surveillance of NH staff and visitors reduces COVID-19 incidence rates in NH residents and allows to reduce visiting restrictions.

**Methods:**

This pilot study at the beginning of the COVID-19 pandemic compared a surveillance approach of regular, twice-weekly voluntary PCR testing of health-care workers (HCW) and visitors in interventional NH (INH) with a setting without regular testing in control NH (CNH). Residents were not tested routinely within this study. Testing was performed in a mobile testing site with same-day result reporting. SARS-CoV-2 incidence among residents in both INH and CNH was the primary endpoint; secondary endpoints being SARS-CoV-2 infection among visitors and HCW in INH.

**Results:**

Two INH and two CNH participated between October and December, 2020. At INH1, 787 tests of HCW and 350 tests of visitors were performed, accounting for 18.1% (*n* = 1930) of visits. At INH2, 78 tests of HCW and 372 tests of visitors were done, i.e., 30.5% (*n* = 1220) of visits. At the two INH 23 HCW and three visitors tested positive for SARS-CoV-2. COVID-19 outbreaks occurred among residents in INH1 (identified through study testing) and in CNH1. Utilization of voluntary testing was low.

**Conclusion:**

In a real-world setting without available rapid testing, voluntary RT-PCR SARS-CoV-2 testing of HCW and visitors does not prevent COVID-19 outbreaks in NH. Complete, non-selective testing for these groups should be instituted before visiting restrictions can be reduced.

**Trial registration:**

The study has been registered at ClinicalTrials.gov with the identifier: NCT04933981.

**Supplementary Information:**

The online version contains supplementary material available at 10.1007/s15010-021-01716-4.

## Introduction

The COVID-19 pandemic continues to cause an unprecedented burden for health-care systems worldwide due to high levels of morbidity and mortality [1]. In particular, residents in nursing homes for the elderly (NH) are a high-risk population for an untoward course of COVID-19 [2, 3]. It is estimated that almost half of all COVID-19 deaths worldwide occurred in NH residents [4]. Outbreaks in NH have led to a case fatality of up to 32% and a sixfold excess mortality compared with the pre-pandemic period [5]. SARS-CoV-2 may be transmitted to NH residents via asymptomatic or oligosymptomatic infected health-care workers (HCW) and visitors [6]. Therefore, at the beginning of the COVID-19 pandemic, visits to NH were largely suspended in Germany with broad psychological and social constraints for NH residents [7]. Surveillance strategies were implemented, but did not prevent COVID-19 outbreaks successfully [8, 9]. PCR testing of HCW and visitors has been suggested a safe approach to prevent outbreaks in NH, because an asymptomatic person with a negative PCR test may not transmit SARS-CoV-2 for up to 72 h post-sampling [10, 11]. However, PCR testing can have a substantial turnaround time from swab to reporting of the result. Besides, testing capacity is often limited [12]. Meanwhile, point-of-care rapid antigen tests (POCT) became available as standard method for entry policy in NH and other facilities, while PCR remains the gold standard for reliable diagnosis of SARS-CoV-2 [13].

At the beginning of the COVID-19 pandemic, when POCT were not available, we hypothesized that offering repeated rapid turnaround PCR surveillance to NH staff and visitors may reduce incidence in NH residents and subsequently allows to reduce visiting restrictions. We addressed this hypothesis by accompanying a regional pilot study of a mobile testing site (MTS) in nursing homes.

## Methods

### Study design

The study compared an approach of regular (i.e., two-to-three times weekly) and voluntary, i.e., non-mandatory, on-site testing of HCW and visitors (interventional nursing homes; INH) with the routine setting without frequent regular testing (control nursing homes; CNH).

Residents were not tested as part of this study. When there was a medical indication for SARS-CoV-2 testing such as symptoms compatible with COVID-19, testing was performed by local health authorities. The pre-specified observational period was planned to span from early October 2020 to mid-December 2020 at maximum.

We evaluated the occurrence of symptomatic SARS-CoV-2 incidence among residents in both INH and CNH as primary endpoint (with an outbreak defined as occurrence of ≥ 1 SARS-CoV-2-infected resident in a timely and situational context). Secondary endpoints were (1) SARS-CoV-2 infections, both asymptomatic and symptomatic, among visitors in INH and (2) SARS-CoV-2 infections, both asymptomatic and symptomatic, among HCW in INH.

We added the following post hoc exploratory descriptive analyses: (1) Ct values in RT-PCR samples to compare sensitivity of SARS-CoV-2 PCR and POCT. A Ct value of 27 in RT-PCR was set as cut-off for secure detection by POCT as described before [14]. (2) Overall mortality, COVID-19-related mortality defined as death while infected with SARS-CoV-2, and excess mortality were assessed by comparing NH mortality data of the same period in the previous year.

### Nursing homes and infection control policy

NH in Cologne were selected for study inclusion by number of residents and willingness to take part, either as control nursing home (CNH) or interventional nursing home (INH). INH and CNH were group-matched according to number of residents and facility size. Four NH participated in the study, each two INH—INH1 with 180 residents and INH2 with 80 residents; and two CNH—CNH1 with 176 residents and CNH2 with 85 residents. In the INHs and CNHs, 335 and 425 permanent personnel were employed and provided care and supporting services for 260 and 261 residents, respectively. We assumed that a dense testing interval of HCW and visitors (two-to-three times weekly) is able to detect a potential SARS-CoV-2 infection early. All entry/access precautions for NH visitors were implemented according to state law [15, 16]. Upon entering a NH, wearing PPE including surgical or FFP2-masks were mandatory for employees and visitors. During the observational period, visits were only allowed for 30 min per day in a visiting area, not in an NH resident’s room. We addressed these restrictions in our tested population as follows: for INH visitors tested SARS-CoV-2 negative via PCR up to 72 h ago, visiting times were expanded from 30 min per day up to 4 h and visits inside the residents’ private rooms were allowed as well as PPE reduction to FFP-masks only, i.e., without full body PPE.

### Mobile testing site (MTS)

The MTS is a vehicle provided and equipped by the University Hospital of Cologne (UHC) as part of the UHC COVID-19 rapid response infrastructure [17].

Every participating HCW and visitor was registered via the “UHC Corona Web Tool” (Healex GmbH, Cologne, Germany), a browser-based smartphone application that, among other functions, records recent history of symptoms and includes an informed consent form for anonymous data utilization [18]. During the login process, personal data are entered, and a QR code is created to register an individual into the UHC electronic patient chart (ePA) (ORBIS®, Dedalus Healthcare Group, Bonn, Germany) and allow automated test result delivery via text message.

**Fig. 1:**
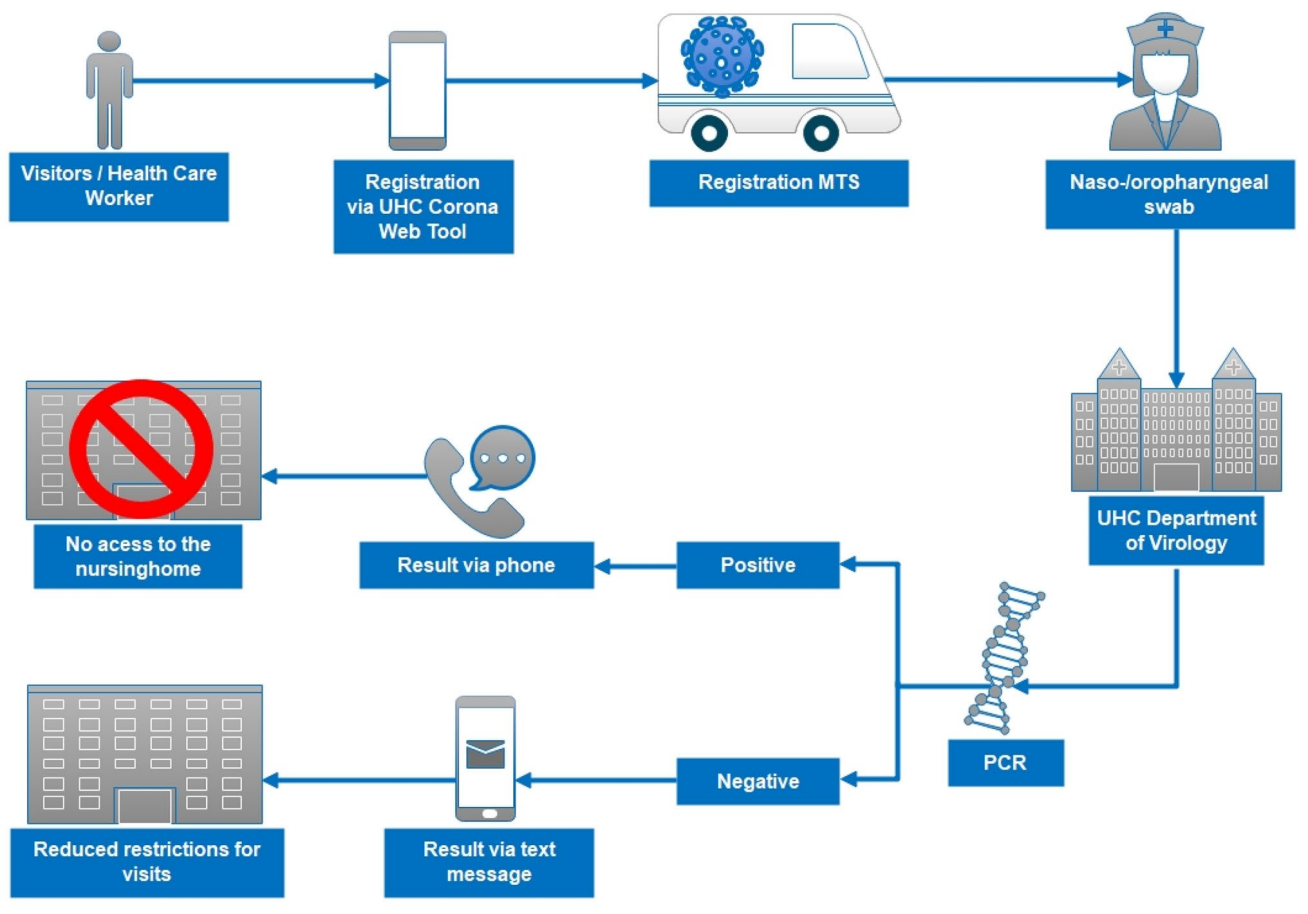
Structure and process of mobile SARS-CoV-2 testing at interventional nursing homes. *MTS* mobile testing site; *PCR* polymerase chain reaction; *UHC* University Hospital of Cologne;

After registration, a combined naso-oropharyngeal (NOP) swab for SARS-CoV-2 detection was performed (Fig. 1).

### Laboratory testing

All samples collected during a given testing day were transferred to the UHC virology laboratory and processed immediately. SARS-CoV-2 RNA detection in combined NOP swabs of asymptomatic individuals was performed with pipette-pool testing (pool size *n* = 10) using the cobas® SARS-CoV-2 test on a cobas® 6800 system (Roche Diagnostics, Mannheim, Germany) [19]. SARS-CoV-2 RNA-positive pools were resolved by testing individual samples. Swabs of individuals with symptoms compatible with COVID-19 were analyzed without pooling using either the cobas® SARS-CoV-2 test, the Xpert® Xpress SARS-CoV-2 assay (Cepheid Europe, Maurens-Scopont, France), or the Alinity m SARS-CoV-2 assay (Abbott, Wiesbaden, Germany) according to the manufacturer’s instructions. These three CE- and IVD-marked assays are dual-target qualitative multiplex real-time PCRs for the detection of SARS-CoV-2 RNA in NOP swab samples. Results of SARS-CoV-2 PCR were available on the same night of the sampling day and were delivered via text message to the participants’ smartphone immediately. In case of a positive result, the affected individual was also called by a physician for further instructions (Fig. 1).

### Data documentation and statistical analysis

Participant data were exported from the “UHC Corona Web Tool” and UHC electronic patient record. Aggregated, thus anonymous, results of NH residents or employees tested by local health authorities were transferred to the study team. Qualitative data were summarized by absolute and relative (%) frequency, quantitative data by median, and interquartile range (IQR). Differences in categorical frequency distributions were only tentatively evaluated using the Chi-square test, since the assumption of independent observations is untenable, while more adequate methods require more data. Figures were created using the open-source python plotting library Matplotlib (https://matplotlib.org/). Data documentation was done in Excel (Microsoft Corp., Redmond, WA, USA), and the statistical analysis was performed with Excel and SPSS Statistics (IBM Corp., Armonk, NY, USA).

### Informed consent and ethical assessment

Informed consent was obtained as part of the registration process. Implementation of the UHC Corona Web Tool for this study was registered with the data privacy software 2B Advice PrIME and approved by the UHC data protection body. This study was approved by the Ethics Committee (No 20-1500_1) of the Medical Faculty of the University of Cologne.

## Results

At the beginning of the study period, local incidence of SARS-CoV-2 in the City of Cologne was 99.4 infections/100,000 inhabitants/week, then rose to a maximum of 227.9 infections/100,000 inhabitants/week on October 30th, subsequently decreased to a lowest level of 129.3 infections/100,000 inhabitants/week on November 27th, and then rose again to 161.8 infections/100.000 inhabitants/week by December 18th, 2020.

During the observational period, 1587 NOP swabs—722 from visitors and 865 from employees of the INH—were performed by the MTS. The mean number of tests per week across all INH was 174.5 (min 136–max 242).

At INH1, 787 tests of HCW and 350 tests of visitors were performed, accounting for 18.1% (*n* = 1930) of visits. At testing, 89 individuals reported symptoms compatible with COVID-19. Sixty visitors and 158 employees were tested more than once during the observational period.

At INH2, 78 tests of employees and 372 tests of visitors were done, accounting for 30.5% (*n* = 1220) of all visits. At testing, 17 individuals reported symptoms compatible with COVID-19. Fifty visitors and eleven employees were tested more than once (Table 1).

**Table 1:** Characteristics of interventional nursing homes (INH1 and INH2) and control nursing homes (CNH1 and CNH2)

Categories	INH1	INH2	CNH1	CNH2
Employees in total, *n*	250	85	315	110
Nursing staff, *n*	120	42	152	55
Single room rate, %	80%	100%	80%	86%
Number of residents, *n*	180	80	176	85
Location of NH	Urban	Suburb	Urban	Suburb
Distribution of residents with COVID-19 at time of outbreak	On several wards		On several wards	
Use of rapid tests (POCT) from	15-Dec-2020	21-Dec-2020	16-Dec-2020	01-Dec-2020
Use of rapid tests (POCT) for visitors available from			24-Dec-2020	15-Dec-2020
Visits in total, *n*	1930	1220	1596	2098

Tests within surveillance study^a^	INH1	INH2
Employees, *n*	787	78
External employees, *n*	12	12
Visitors, *n*	350	372
SARS-CoV-2-positive employees, *n*	22	1
SARS-CoV-2-positive external employees, *n*	0	0
SARS-CoV-2-positive visitors, *n*	3	0

*NH* nursing home; *INH* interventional nursing homes; CNH, control nursing homes; POCT, point-of-care test

^a^numbers only for tests within the surveillance study, tests by local health authorities excluded

### Test results

In total, three visitors and 23 employees tested positive for SARS-CoV-2, i.e., three visitors and 22 HCW of INH1 and one HCW of INH2 (Table S1).

Based on data provided by local health authorities including separate and concurrent testing, in the two CNH 25 employees and 20 residents and in the INH, 63 employees and 76 residents tested SARS-CoV-2 positive (Table S2).

**Fig. 2:**
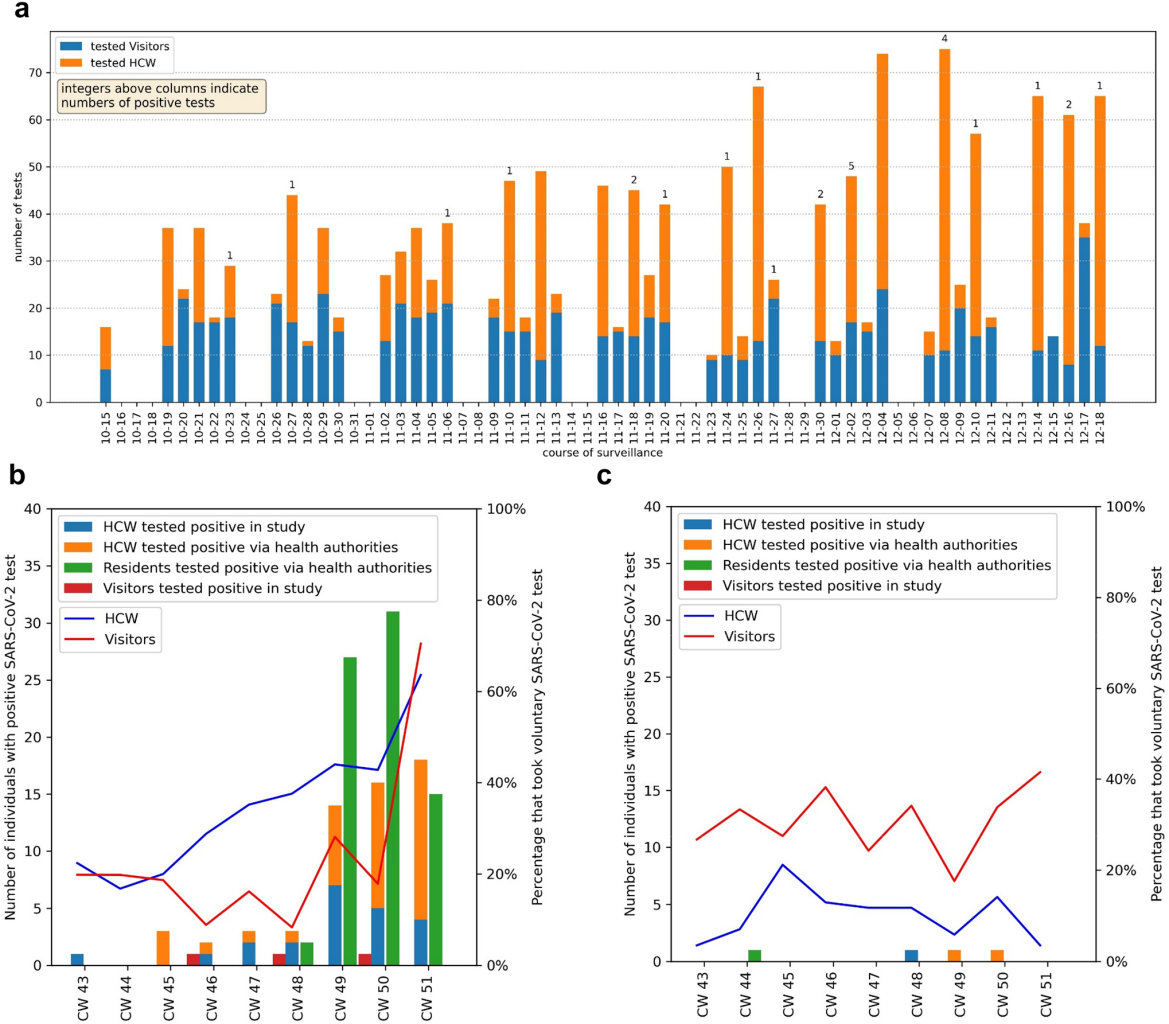
**a** Timeline of testing and positive results at interventional nursing homes. **b** SARS-CoV-2 PCR test results and voluntary utilization of testing in INH1. **c** SARS-CoV-2 PCR test results and voluntary utilization of testing in INH2. *CW* calendar week; *HCW* health-care worker; *INH* interventional nursing home

One outbreak each were first detected at INH1 by the MTS (Fig. 2a–c) and at CNH1 by local health authorities.

### Mortality

Sixty-three (12.1%) NH residents died during the observational period, compared to 54 (10.4%) during the period in the previous year (Fig. S2a–b). All-cause mortality in the INH was 15% (39/260) and COVID-19-related mortality was 8.8% (23/260), all of them in INH1, during the study period. All-cause mortality in the CNH was 9.2% (24/261), and COVID-19-related mortality was 1.5% (4/176), all of them in CNH1 (Table 2).

**Table 2:** Mortality (number of deaths) in INH and CNH from 2017 until 2020

	2017, *n*	4th Quarter 2017, *n*	2018, *n*	4th Quarter 2018, *n*	2019. *n*	4th Quarter 2019. *n*	2020, *n*	4th Quarter 2020, *n*	COVID-19-related mortality, *n*
INH1	45	15	67	8	45	9	55	28	23
INH2	17	8	29	5	29	8	40	11	0
INH total	62	23	96	13	74	17	95	39	23
CNH1	53	10	63	15	71	23	59	17	4
CNH2^a^					14	14		7	0
CNH total	53	10	63	15	85	37	59	24	4
							*p* < 0.05^b^	*p* < 0.05^b^	

^a^Data of CNH2 are not evaluable until third quarter of 2019 due to construction work and hence varying numbers of residents and staff numbers

^b^Mortality compared to previous year using the Chi-square test

### Detection of SARS-CoV-2 in RT-PCR versus POCT

**Fig. 3:**
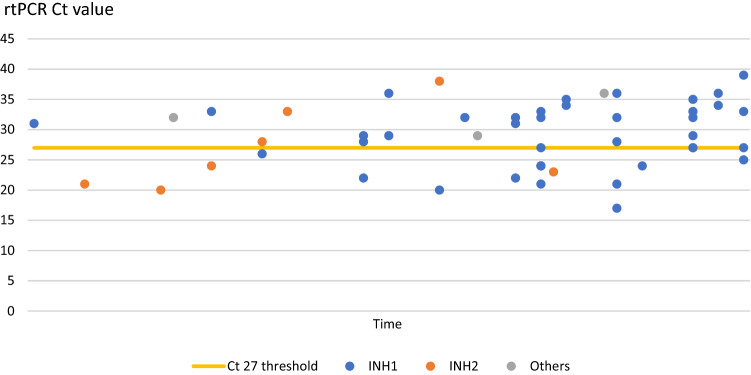
Ct values of all positive SARS-CoV-2 test results (including-INH participants and sequentially tested participants). *CNH* control nursing home; *Ct* cycle threshold; *INH* interventional nursing home; *RT-PCR* reverse-transcriptase polymerase chain reaction; others include SARS-CoV-2 swabs performed by the MTS outside the study population during the observational period

During the conduct of this study, the MTS detected 53 positive SARS-CoV-2 RT-PCR from NOP swabs. This included NH employees who were tested sequentially, i.e., more than once. Further virological analysis showed Ct values > 27 in 34 and ≤ 27 in 19 samples, respectively. Of those 53 tests, 26 were first-time positive test results of individual visitors (*n* = 3) and employees (*n* = 23) (Tab. S1). Evaluation of Ct values in this group revealed Ct values > 27 in 13 and ≤ 27 in 13 samples, respectively. Of the SARS-CoV-2 infected NH staff, 12 had Ct values > 27 at the time of their first test, meaning that detection by commercially available POCT would not have been reliable due to limited sensitivity. (Fig. 3).

## Discussion

This pilot study compared two approaches for SARS-CoV-2 surveillance of NH: an interventional approach with frequent voluntary, i.e., non-mandatory, SARS-CoV-2 testing of HCW and visitors versus a control approach without any specific surveillance. We underline the real-world setting in which the study was performed, meaning that in many places, routine SARS-CoV-2 testing was not available and local health-care authorities were not prepared to support high-risk settings such as NH which is still the case to date in many countries with resource-limited health-care settings [20, 21].

Both approaches complied with local pandemic law regulations, while the first approach allowed partial loosening of certain visiting policies for visitors as described. The INH and CNH were comparable regarding their size and location. The MTS provided an example of resource allocation for regular non-mandatory testing as part of public health measures in response to the COVID-19 pandemic in NH [22].

With regards to the primary endpoint, SARS-CoV-2 incidence of NH residents, our pilot study failed to demonstrate a significant benefit of the interventional surveillance approach over the control strategy. However, surveillance with regular non-mandatory testing also identified solitary cases of SARS-CoV-2 infection among HCW leading to immediate isolation of the affected individuals and subsequently may have avoided even more SARS-CoV-2 infections in NH.

Two observed outbreaks among residents occurred in INH1 and CNH1, both being facilities with more than 100 residents. Larger facility size has been described as risk factor for SARS-CoV-2 outbreaks with a higher number of HCW and visitors amplifying the risk for transmission [23].

Utilization of testing was low in our study, probably due to the voluntary approach. A systematic surveillance study in a congregate housing setting modeled a 154% increase of SARS-CoV-2 detection when frequent regular voluntary testing upon invitation was performed compared with random voluntary testing only [24]. Interestingly, in our study, a higher utilization of tests by HCW was observed when incidence among residents increased in INH1 in the outbreak during the study period.

In our study, positive SARS-CoV-2 tests were far more frequent among HCW than among visitors. We believe that mandatory and regular (e.g., twice weekly) RT-PCR SARS-CoV-2 testing is crucial for HCW working in congregated housing settings and needs to be addressed adequately by policy makers and NH operators.

The social situation in NH has dramatically changed during the pandemic [25]. Prohibition of visits of residents leads to substantial psychological sequelae [26, 27]. Based on our observations, visitors do not seem to represent an important transmission source of SARS-CoV-2 compared to HCW. We hypothesize that our approach—if made mandatory for visitors—may decrease visiting restrictions and may thus lead to increased emotional well-being through ensuring a minimum of social contacts without favoring the occurrence of SARS-CoV-2 outbreaks. Studies assessing a socio-psychological benefit for nursing home residents may further elucidate any such effect.

### Mortality

Despite the two outbreaks that occurred, there was no increased mortality compared with previous years across all NH in our study. However, in INH1—with one SARS-CoV-2 outbreak—a three times higher mortality in the fourth quarter of 2020 was observed. Nevertheless, COVID-19-related mortality across all NH in our study was slightly lower than in other studies in NH during the same period [28].

An association of increased mortality in NH residents after SARS-CoV-2 infection of employees was demonstrated with an adjusted mortality incidence rate ratio for death per infected staff member of 1.17 [29]. This underlines the impact of infected HCW on viral spread in NH. We found more SARS-CoV-2-positive subjects among HCW than among visitors, suggesting that mainly employees with close contact to residents are a risk for transmission to residents, whereas visitors may not be drivers of infection.

### SARS-CoV-2 RT-PCR vs. POCT

Meanwhile, the development of POCT has progressed and wide-spread availability is now ensured. Leading infection control authorities recommend POCT use for SARS-CoV-2 testing to support regular screening of staff and outbreak investigations [2, 30]. POCT have become the diagnostic standard for screening due to availability, lower cost, and shorter time-to-result.

However, the sensitivity of POCT remains low for surveillance purposes, since mostly asymptomatic individuals are screened and can be as low as 41.2% in a real-world setting [31]. Subjects tested without symptoms compatible with COVID-19 reduce pre-test probability of POCT and contribute to a low positive predictive value. False-negative POCT rates will rise during times of high incidence of COVID-19 making PCR the more secure method for effective mitigation of SARS-CoV-2 transmission in NH [13].

We detected 34 individuals (64.2%) with positive SARS-CoV-2 RT-PCR with a Ct value higher than 27. Of those, 13 were staff or visitors of NH without previous knowledge of their SARS-CoV-2 infection who probably would not have been detected by POCT and subsequently could have infected NH residents despite adhering to infection control policies.

These results suggest a rather low detection rate for POCT [32, 33]. We propose frequent regular RT-PCR testing for SARS-CoV-2 to maintain the gold standard for HCW surveillance to secure best available protection of an at-risk population for severe COVID-19 and death.

### Limitations

An intensified testing strategy arouses suspicion of reporting bias with a higher infection rate due to increased detection of asymptomatic individuals. Our pilot study involved only four NH leading to a small number of observations. Thus, the statistical power to detect relevant differences between intervention and control strategies was expectedly deficient. Of note, voluntary testing may lead to self-selection bias. Besides, due to regulatory and ethical reasons, we were not able to take swabs, neither voluntary nor mandatory, from NH residents. Our study required use of a smartphone to get tested. Smartphones are not widely distributed among the elderly and their relatives which may also limit wider implementation of our approach.

### Future prospects

Since December 2020, a number of anti-SARS-CoV-2 vaccines have been licensed in Europe. Vaccination strategies differ by country and region and residents of NH and HCW represent a high priority group; therefore, large numbers of residents of NH are already vaccinated against SARS-CoV-2 [34]. Nonetheless, surveillance of NH remains important, since vaccines may not prevent COVID-19 in all subjects, especially in the elderly population due to immunosenescence [35, 36]. Even after vaccination of residents, outbreaks in NH still occur, although with less impact in terms of disease severity and mortality, but with a potential high impact as drivers of infection [37]. Our findings remain relevant if emerging immune-escape SARS-CoV-2 variants-of-concern with the potential of vaccine-derived humoral immune escape cause infections in NH in the future [38, 39]. Our study can be considered an innovative pilot project to assess feasibility of systematic SARS-CoV-2 testing with the goal of reducing restrictions under real-life conditions in the context of the COVID-19 pandemic in Germany [40].

First, we show that despite offering regular on-site PCR-based surveillance testing, NH outbreaks can occur. Second, we highlight that utilization of tests remains low and conclude that especially HCW may introduce infections into the facilities. Third, we encourage non-selective, i.e., mandatory, surveillance testing in NH settings, so any SARS-CoV-2 infection can be rapidly detected among employees to prevent outbreaks efficiently. We generated an initial knowledge base and thus a potential template for larger surveillance studies in NH. This may support scientists and public health specialists in developing concepts for future pandemics and encourage policy makers to allocate testing resources efficiently.

## Supplementary Information

Below is the link to the electronic supplementary material.

Supplementary file1 (DOCX 166 KB)
