# Supplementary material for: Mobile PCR-based surveillance for SARS-CoV-2 to reduce visiting restrictions in nursing homes during the COVID-19 pandemic: a pilot study
Source: Infection. 2021 Oct 20;50(3):607–16. doi: 10.1007/s15010-021-01716-4 (PMC8527812; doi:10.1007/s15010-021-01716-4)
Supplement: Supplementary file 1 — Supplementary file1 (DOCX 166 KB) [file 15010_2021_1716_MOESM1_ESM.docx]

## **Mobile** **PCR-based surveillance for SARS-CoV-2 to reduce visiting restrictions in nursing homes during the COVID-19 pandemic: a pilot study**

Jannik Stemler, MD^1,2,3#^, Theresa Kramer^1,2#^, Vassiliki Dimitriou, PhD^1,2,3^, Prof. Ulrike Wieland, MD^4^, Sofie Schumacher, MD^1,2,3^, Rosanne Sprute, MD^1,2,3^, Max Oberste, PhD^5^, Prof. Gerhard Wiesmüller, MD^6^, Harald Rau, PhD^7^, Sally Pieper^1,2^, Ullrich Bethe, MD^1,2^, Prof. Clara Lehmann, MD^8^, Prof. Martin Hellmich, PhD^5^, Prof. Florian Klein, MD^4^, Georg Langebartels, MD^9^, Prof. Oliver A. Cornely, MD^1,2,3,10^

**Affiliations**

1. University of Cologne, Faculty of Medicine and University Hospital Cologne, Department I of Internal Medicine, Excellence Center for Medical Mycology (ECMM), Cologne, NRW, Germany
2. University of Cologne, Faculty of Medicine and University Hospital Cologne, Chair Translational Research, Cologne Excellence Cluster on Cellular Stress Responses in Aging-Associated Diseases (CECAD), Cologne, NRW, Germany
3. German Centre for Infection Research (DZIF), Partner Site Bonn-Cologne, Cologne, NRW, Germany
4. Institute of Virology, University of Cologne, Faculty of Medicine and University Hospital Cologne, NRW, Germany
5. Institute of Medical Statistics and Computational Biology (IMSB), University of Cologne, Faculty of Medicine and University Hospital Cologne, NRW, Germany
6. Department of Public Health, City Council of Cologne, NRW, Germany
7. Department of Social Affairs, Health and Environment, City Council of Cologne, NRW, Germany
8. University of Cologne, Faculty of Medicine and University Hospital Cologne, Department I of Internal Medicine, Cologne, NRW, Germany
9. University of Cologne, Faculty of Medicine and University Hospital Cologne, Department for Clinical affairs and Crisis management, Cologne, NRW, Germany
10. University of Cologne, Faculty of Medicine and University Hospital Cologne, Clinical Trials Centre Cologne (ZKS Köln), Cologne, NRW, Germany

^#^Both authors contributed equally

| *Author* | *ORCID ID* |
| --- | --- |
| Jannik STEMLER | https://orcid.org/0000-0001-9152-2469 |
| Theresa KRAMER | https://orcid.org/0000-0001-5187-6510 |
| Vassiliki DIMITRIOU |  |
| Ulrike WIELAND | https://orcid.org/0000-0003-3480-4413 |
| Sofie SCHUMACHER | https://orcid.org/0000-0002-8805-1421 |
| Rosanne SPRUTE | https://orcid.org/0000-0003-2457-6437 |
| Max OBERSTE | https://orcid.org/0000-0002-1224-5385 |
| Gerhard WIESMÜLLER |  |
| Harald RAU | https://orcid.org/0000-0001-6681-2288 |
| Sally PIEPER |  |
| Ullrich BETHE | <https://orcid.org/0000-0001-9979-1671> |
| Clara LEHMANN | [https://orcid.org/0000-0002-7042-1578](https://orcid.org/0000-0002-7042-1578?lang=en) |
| Martin HELLMICH | https://orcid.org/0000-0001-5174-928X |
| Florian KLEIN | https://orcid.org/0000-0003-1376-1792 |
| Georg LANGEBARTELS |  |
| Oliver A. CORNELY | https://orcid.org/0000-0001-9599-3137 |

**Corresponding author:**

Prof. Oliver A. Cornely, FACP, FIDSA, FAAM, FECMM

University of Cologne

Cologne Excellence Cluster on Cellular Stress Responses in Aging-Associated Diseases (CECAD)

Herderstrasse 52

50931 Cologne, Germany

Phone: +49 (0) 221 478 85523

Fax: +49 (0) 221 478 1435276

E-mail: [Oliver.Cornely@uk-koeln.de](mailto:Oliver.Cornely@uk-koeln.de)

## Supplementary Material

**Table S1: SARS-CoV-2 positive tested staff / visitors, individual clinical characteristics, and Ct values**

|  | Test date | Result | Date of Result | clinical characteristics | Lower RT-PCR Ct value | RT-PCR Ct≤27 |
| --- | --- | --- | --- | --- | --- | --- |
| INH 1 | 10.11.2020 | POS | 11.11.2020 | Staff asymptomatic | 26,40 | Yes |
| INH 1 | 18.12.2020 | POS | 18.12.2020 | Staff asymptomatic, direct contact | 22,00 | Yes |
| INH 1 | 23.10.2020 | POS | 23.10.2020 | Staff asymptomatic | 31,29 | No |
| INH 1 | 18.11.2020 | POS | 19.11.2020 | Staff asymptomatic | 27,09 | No |
| INH 1 | 02.12.2020 | POS | 02.12.2020 | Staff with symptoms: Cough for one day | 26,72 | Yes |
| INH 1 | 14.12.2020 | POS | 15.12.2020 | Staff with symptoms: Cough for one day | 27,23 | Yes |
| INH 1 | 30.11.2020 | POS | 01.12.2020 | Staff asymptomatic, direct contact | 22,04 | Yes |
| INH 1 | 30.11.2020 | POS | 01.12.2020 | Staff asymptomatic | 30,10 | No |
| INH 1 | 02.12.2020 | POS | 02.12.2020 | Visitor asymptomatic, direct contact | 21,32 | Yes |
| INH 1 | 26.11.2020 | POS | 26.11.2020 | Staff with symptoms: sore throat and headache since today, direct contact | 31,56 | No |
| INH 2 | 27.11.2020 | POS | 27.11.2020 | Staff asymptomatic, previously tested with unclear test result | 26,50 | No |
| INH 1 | 10.12.2020 | POS | 10.12.2020 | Staff with symptoms: Cough and fever for one day | 20,27 | Yes |
| INH 1 | 08.12.2020 | POS | 09.12.2020 | Staff with symptoms: Fever for one day, direct contact | 20,20 | Yes |
| INH 1 | 02.12.2020 | POS | 02.12.2020 | Staff asymptomatic, direct contact | 32,09 | No |
| INH 1 | 02.12.2020 | POS | 02.12.2020 | Staff with symptoms: Fever, headache, aching limbs for one day, direct contact | 33,16 | No |
| INH 1 | 08.12.2020 | POS | 09.12.2020 | Staff with symptoms: Sore throat, headache, diarrhoea for one day, direct contact | 32,40 | No |
| INH 1 | 16.12.2020 | POS | 16.12.2020 | Staff asymptomatic, direct contact | 33,68 | No |
| INH 1 | 18.11.2020 | POS | 18.11.2020 | Staff with symptoms: Migraine with rhinitis and vomiting for one day | 27,51 | No |
| INH 1 | 18.12.2020 | POS | 18.12.2020 | Staff asymptomatic, direct contact | 24,00 | Yes |
| INH 1 | 02.12.2020 | POS | 03.12.2020 | Visitor asymptomatic | 23,50 | Yes |
| INH 1 | 06.11.2020 | POS | 06.11.2020 | Staff with symptoms: headache since today | 32,65 | No |
| INH 1 | 08.12.2020 | POS | 09.12.2020 | Staff with symptoms: Cough for one day, direct contact | 35,80 | No |
| INH 1 | 24.11.2020 | POS | 24.11.2020 | Staff with symptoms: Sore throat, chills, cough for one day | 16,03 | Yes |
| INH 1 | 08.12.2020 | POS | 09.12.2020 | Staff with symptoms: Fever for one day, direct contact | 23,70 | Yes |
| INH 1 | 20.11.2020 | POS | 21.11.2020 | Visitor asymptomatic | 28,82 | No |
| INH 1 | 02.12.2020 | POS | 02.12.2020 | Staff asymptomatic, direct contact | 23,50 | Yes |

**Table S2: SARS-CoV-2 testing results in INH and CNH**

|  | | **SARS-COV 2 RT-PCR tests among residents** | |  |
| --- | --- | --- | --- | --- |
|  |  | **Positive** | **Negative** |  |
| Surveillance strategy | **CNH** | 20 | 241 |  |
|  | **INH** | 76 | 180 |  |
|  | **Total** | 96 | 421 | *p*<0.001^a^ |

Abbr.: CNH, control nursing home; INH, interventional nursing home

^a^using Chi²-test

**Figure S2a: Annual mortality in NH from 2017 to 2020**


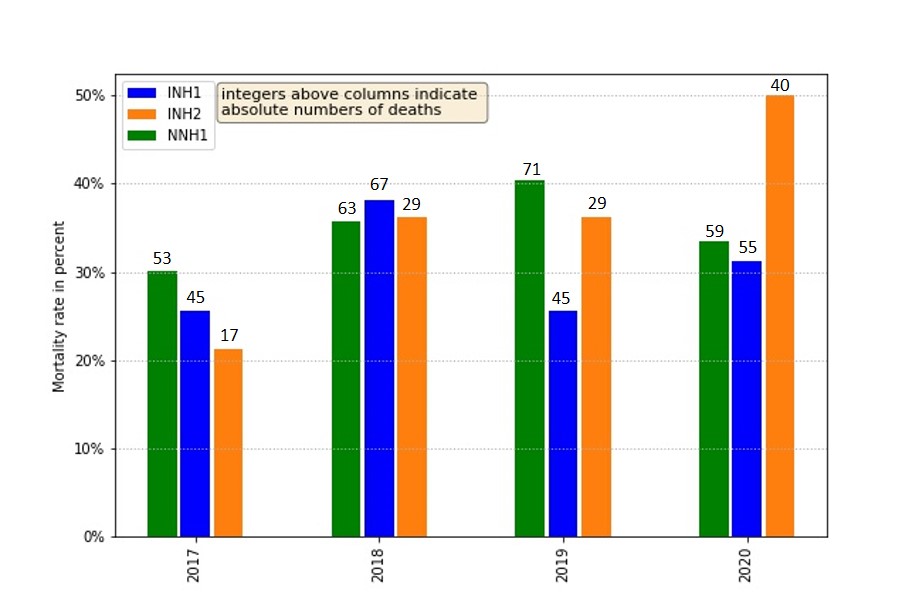


**Figure S2b: Mortality in fourth quarter in NH from 2017 until 2020**


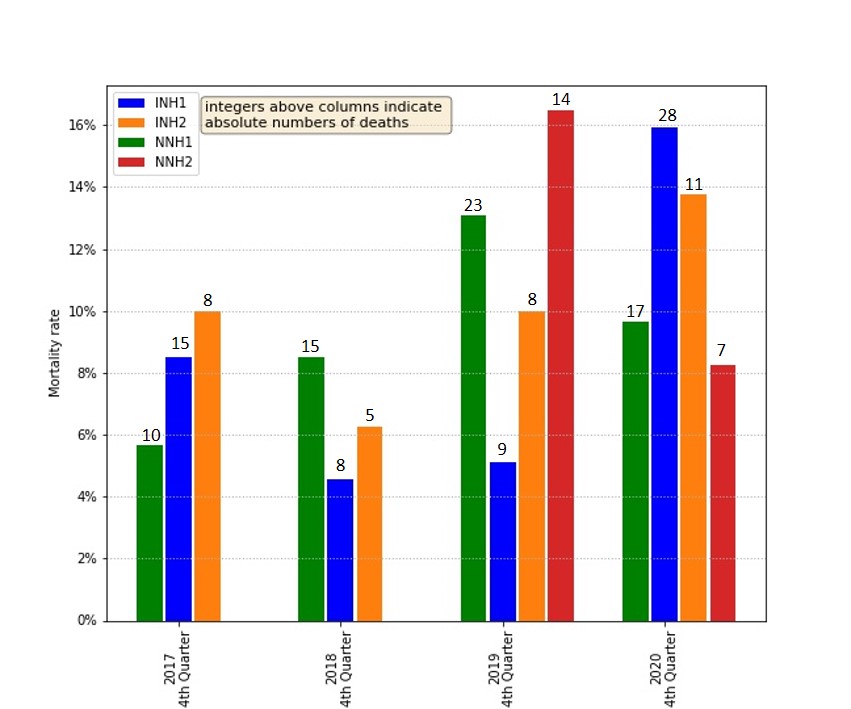


*mortality data of NNH2 not available due to re-opening of this facility in 2019
